# A Novel Machine Learning Aided Antenna Selection Scheme for MIMO Internet of Things

**DOI:** 10.3390/s20082250

**Published:** 2020-04-16

**Authors:** Wannian An, Peichang Zhang, Jiajun Xu, Huancong Luo, Lei Huang, Shida Zhong

**Affiliations:** College of Electronics and Information Engineering, Shenzhen University, Shenzhen 518060, China; anwannian2018@email.szu.edu.cn (W.A.); xujiajun2017@email.szu.edu.cn (J.X.); huancongluo@163.com (H.L.); lhuang@szu.edu.cn (L.H.); shida.zhong@szu.edu.cn (S.Z.)

**Keywords:** MIMO, antenna selection (AS), Internet of Things (IoT), machine learning, multi-label convolution neural network

## Abstract

In this article, we propose a multi-label convolution neural network (MLCNN)-aided transmit antenna selection (AS) scheme for end-to-end multiple-input multiple-output (MIMO) Internet of Things (IoT) communication systems in correlated channel conditions. In contrast to the conventional single-label multi-class classification ML schemes, we opt for using the concept of multi-label in the proposed MLCNN-aided transmit AS MIMO IoT system, which may greatly reduce the length of training labels in the case of multi-antenna selection. Additionally, applying multi-label concept may significantly improve the prediction accuracy of the trained MLCNN model under correlated large-scale MIMO channel conditions with less training data. The corresponding simulation results verified that the proposed MLCNN-aided AS scheme may be capable of achieving near-optimal capacity performance in real time, and the performance is relatively insensitive to the effects of imperfect CSI.

## 1. Introduction

In recent years, as an emerging communication paradigm, the Internet of Things (IoT) has drawn researchers’ substantial attention due to its capability of providing massive low cost connections for a wide range of smart applications [1]. In practical IoT systems, the devices are usually sensitive to the power constraint, which may significantly limit the throughput and coverage performance of the overall IoT systems. In this case, the novel concept of multiple-input-multiple-output (MIMO) communications was introduced in IoT systems for the sake of offering higher data throughput, wider signal coverage and lower power consumption [2,3,4]. However, classical MIMO systems are usually equipped with multiple antennas, which are linked to the same number of radio-frequency (RF) chain pairs. This not only imposes high computational complexity, but also requires multiple RF chains in the communication process, which greatly raises the hardware costs and may increase the power consumption. As a remedy, the antenna selection (AS) technologies were proposed for the sake of reducing MIMO complexity and hardware costs, while retaining the advantages of MIMO communication systems.

Generally, there are two types of AS schemes, namely the norm-based AS (NBAS) and capacity-based AS (CBAS). The idea of NBAS criterion is to select the antennas subset associated with the highest channel gain to obtain the maximum equivalent signal-to-noise ratio (SNR) [5,6,7], while the CBAS schemes select the antennas subset associated with the highest channel capacity [7,8]. Usually, AS-related research is based on the assumption of independent MIMO channels, while in practice, the physical size of MIMO facilities and devices is often limited. Therefore, as the number of antennas used for transmitting and/or receiving increases, the correlation between antennas may increase as well. In this case, the NBAS may experience severe performance loss [9,10,11]. This is because that in correlated MIMO environments, antennas associated with similar channel gain are usually closely located. In this case, antennas with higher channel gains may be selected by NBAS and may lead to even higher inter-antenna correlation [12], thus causing MIMO performance loss. On the other hand, CBAS algorithms are based on the capacity performance of the selected antennas, which would not cause higher inter-antenna correlation after AS process [13]. Therefore, in correlated MIMO systems, especially in massive MIMO systems, the CBAS criterion is usually considered. However, the optimal performance of CBAS is usually achieved by ergodic search-based methods, which may significantly increase the computational complexity in MIMO systems. Several works focused on sub-optimal CBAS criteria with lower computational complexity. For example, refs. [14,15,16] proposed incremental successive selection algorithm (ISSA) to seek out optimal set of transmit and receive antennas, which started with an empty antenna set, and then in each step, it added the antenna that contributed the most to the system’s capacity to the AS set. Refs. [17,18] studied the genetic algorithms (GA) for joint transmit and receive AS (JTRAS). Specifically, ref. [17] proposed a simplified GA using bit string and basic binary operation to select joint transmit and receive antennas based on the maximum instantaneous capacity. Additionally, the JTRAS based on priority GA is proposed in [18], which modified the two main steps of crossover and mutation of conventional GA, then inserted the priority mechanism into the modified GA. Furthermore, ref. [13] exploited a cross entropy optimization (CEO)-based AS algorithm, which was shown to be a global random search process with fast convergence performance. In addition, a G-circles method based on the characteristics of matrix determinant and geometric analysis was also analyzed for AS in [19]. All of the these above mentioned AS methods are capable of achieving near-optimal CBAS performance, and relatively reducing the computational complexity of AS compared to the optimal ergodic search-based methods. However, it should be noticed that these AS algorithms are purely mathematical optimization driven algorithms, which still require certain realtime computation resources, and thus cause high power consumption and latency in MIMO IoT systems.

Recently, an increasing number of works started to focus on applying machine learning (ML) technologies in a wide range of communication applications due to the fact that ML is capable of transfering the conventional mathematical optimization problems into data-driven problems for achieving lower online realtime computational complexity [20,21,22]. More specifically, it has been well recognized that the ML schemes, i.e., convolutional neural network (CNN), exhibit excellent performance in image processing, especially in classification problems [23,24,25,26]. Based on the intuitive thought that a channel state information (CSI) matrix in MIMO communications can be regarded as a two-dimensional image, it is promising to similarly consider the ML schemes in MIMO communications as in image processing. In particular, ref. [20] applied ML technique to wireless communication systems, and interpreted the AS for MIMO communications to multi-class classification learning. Ref. [20] provided insights into the potential of introducing the concept of ML into wireless communications, and verified the feasibility of the ML-based AS algorithms by comparing the performance of the learning-based AS using K-nearest neighbors (KNN) and multi-class support vector machine (SVM) schemes to the conventional mathematical optimization-based AS methods. Moreover, ref. [21] employed the KNN and multi-class SVM classification algorithms for the transmit AS in untrusted relaying networks, where the corresponding numerical results showed that compared to conventional AS schemes, ML-based schemes were capable of achieving nearly the same security performance with less computation overhead. Additionally, ref. [22] proposed the LeNet model for receiving AS schemes, which is a single-label multi-class classification CNN model. The proposed method used convolutional structure to extract the rich features from the channel matrices, and numerical experimental results showed that the LeNet model-based AS outperformed the state-of-the-art baselines. However, it is worth mentioning that the above mentioned ML-based AS methods only considered in small-scale and independent MIMO channel conditions. With the increase of the antenna scale under the correlated MIMO channel conditions, the complexity of these multi-class classification learning schemes may increase with degraded performance.

Against this background, the novel contribution of this work is that we propose a multi-label convolutional neural network (MLCNN)-aided CBAS algorithm for correlated MIMO systems by exploiting the advantages of the ML schemes. First, in contrast to the conventional single-label multi-class classification ML schemes, our proposed MLCNN-based AS scheme may greatly reduce the length of training labels in the case of multi-antenna selection and significantly improve the prediction accuracy of the trained MLCNN model under correlated large-scale MIMO channel conditions with less training data. Secondly, different from the conventional CNN-based ML schemes, the pooling operation is removed from our proposed MLCNN for the sake of fully extracting the characteristics of the MIMO CSI. The corresponding simulation results verified that the proposed MLCNN-aided AS scheme may be capable of achieving near-optimal capacity performance in real time, and the performance is relatively insensitive to the effects of imperfect CSI.

The following notational conventions are adopted throughout our discussions. Boldface capital and lower-case letters stand for matrices and vectors respectively, while ${\left(\cdot \right)}^{T}$ and ${\left(\cdot \right)}^{H}$ represent the transpose operator and conjugate transpose operator, respectively. |h| denotes the magnitude of complex value *h*. max{H} and min{H} denote the element with the maximum value and the element with the minimum value in all elements of matrix H, respectively. Additionally, the M×M identity matrix is denoted by $I_{M}$, H(i,j) is the element in *i*-th column and *j*-th row of matrix H.

The rest of this contribution is organized as follows. Section 2 describes the MIMO wireless communication system model, while our proposed MLCNN-based transmitter AS is detailed in Section 3. The simulation results and discussion are shown in Section 4, while our conclusions are given in Section 5.

## 2. System Model

We consider an end-to-end MIMO wireless communication system, where the transmitter is equipped with $N_{T}$ transmit antennas (TAs) associated with $L_{t}$ ($L_{t}\leq N_{T}$) available RF chains and the receiver is configured $N_{R}$ receive antennas (RAs) associated with the same number of RF chains over an uplink narrowband flat correlated Rayleigh fading channel. We only consider the correlation at the transmitter and assume that there is enough scatterers at the receiver. The corresponding full MIMO channel matrix $H\in C^{N_{R}\times N_{T}}$ may be expressed as

(1) $$\begin{array}{c} H=GR_{T}^{1/2} \end{array}$$

where $G\in C^{N_{R}\times N_{T}}$ is the channel matrix of which the elements are independent identically distributed (i.i.d) and obeys Gaussian random distribution of CN(0,1), $R_{T}\in C^{N_{T}\times N_{T}}$ denotes channel spatial correlation matrix at the transmitter, and we adopt an exponential model of it [11], the calculation formula of the model element is as follows

(2) $$\begin{array}{c} R_{T}\left(l1,l2\right)=\rho_{t}^{\left|l1-l2\right|}\in \left[0,1\right);\;l1,l2=1,\cdots ,N_{T} \end{array}$$

where $corr_{t}$ denotes spatial correlation coefficient between antennas at transmitter. Under the above assumptions, our received signal $y\in C^{N_{R}\times 1}$ may be expressed by [27]

(3) $$\begin{array}{c} y=Hx+v \end{array}$$

where $x\in C^{N_{T}\times 1}$ denotes the transmitted signal, $v\in C^{N_{R}\times 1}$ is denotes as the additive white Gaussian noise (AWGN) obeying the distribution of $\mathrm{CN}(0,a_{n}^{2})$.

## 3. CBAS Aided MIMO

It was mentioned in Section 1 that in correlated MIMO systems, CBAS is usually preferred since it is insensitive to the MIMO channel correlation. For a MIMO system equipped with $N_{T}$ TAs and $N_{R}$ RAs, the system’s MIMO channel capacity may be expressed as [28]

(4) $$\begin{array}{c} C=\log_{2}\left[\det\left(I_{N_{R}}+\frac{p}{N_{T}\cdot a_{n}^{2}}HH^{H}\right)\right] \end{array}$$

where *p* denotes the total transmit power. Let us assume that the CSI is known at the transmitter, then the optimal CBAS may be performed at the transmitter side through selecting $L_{t}$ TAs from the total of $N_{T}$ TAs by

(5) $$\begin{array}{c} H_{max}=\underset{s_{m}\in S}{\arg\max}\left\{\log_{2}\left[\det\left(I_{N_{R}}+\frac{p}{N_{T}\cdot a_{n}^{2}}H_{s_{m}}H_{s_{m}}^{H}\right)\right]\right\} \end{array}$$

where $H_{s_{m}}\in C^{N_{R}\times L_{t}}$ is the subset of full channel matrix $H\in C^{N_{R}\times N_{T}}$, $S=\{s_{1},s_{2},\cdots ,s_{m},\cdots ,s_{\lambda }\}$ represents all possible combinations of TAs with λ=NTLt. The optimal solution $H_{max}\in C^{N_{R}\times L_{t}}$ of CBAS is usually achieved by ergodic capacity comparison of all possible combinations, while the computational complexity may dramatically increase as the number of antennas increases. Several suboptimal CBAS algorithms [12,13,14,15,16,17,18,19] were proposed for the sake of reducing the computational complexity at the cost of certain performance loss. Additionally, for the critical delay and speed requirements of the 5G networks, the online realtime computational complexity of the above mentioned suboptimal CBAS methods is still considered to be relatively high. Therefore, we propose a new MLCNN-aided CBAS algorithm for the sake of reducing the online AS processing time.

### 3.1. Proposed MLCNN

**Figure 1:**
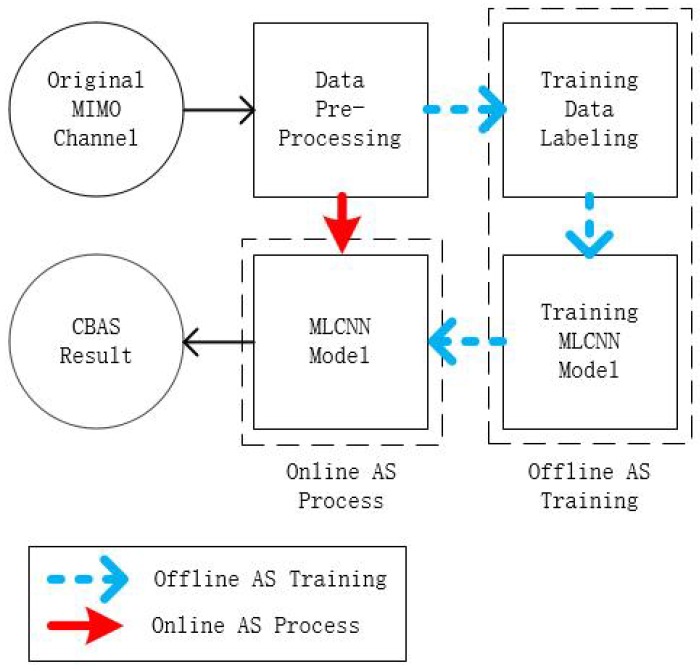
The block diagram of the MLCNN-aided CBAS.

The block diagram of the proposed MLCNN algorithm is depicted in Figure 1, where it may be seen that the MLCNN scheme is generally consisted of three operation phases, namely the data pre-processing, offline AS training and online AS process. More specifically, the simple yet efficient data pre-processing is to ensure the validity of the input data for both offline AS training process and online AS decision process. The offline AS training process is adopted for MLCNN model training, while the online AS process is used for realtime AS decision making. It worths mentioning that the AS training process may be of high computational complexity. However, the training is an offline process and imposes no online realtime delay to the overall system. Therefore, the proposed MLCNN is capable of reducing AS delay since the overall delay is only imposed by a simple data preprocessing and an efficient online AS process. Now let us detail our proposed MLCNN-aided CBAS.

#### 3.1.1. Data Pre-Processing

It was recognized that data normalization may significantly affect the performance of the system when the CNN-based ML schemes are used for recognition and selection purposes [29,30]. In this case, data pre-processing becomes a necessity to normalize the original data for further process, which may help to reduce both the data selection errors and the calculation time during the training process. This is because that data normalization is capable of narrowing the range of data feature differences, while making the network to obtain the ideal weights set in a short time during the training process [29,30]. In the proposed MLCNN-aided CBAS scheme, the complex-valued full MIMO channel of $H\in C^{N_{R}\times N_{T}}$ is adopted as the samples for the training process. Since in CNN the training samples must be real-valued data, the complex-valued full MIMO channel matrix $H\in C^{N_{R}\times N_{T}}$ is firstly pre-processed and normalized by the following three steps:

- Generate *M* full MIMO channel matrices $H\in C^{N_{R}\times N_{T}}$ for training process.
- Take the magnitude of the full MIMO channel matrix elements as $H_{k}\left(i,j\right)=\left|H_{k}\left(i,j\right)\right|$, where $H_{k}\in C^{N_{R}\times N_{T}}$ is the *k*th full channel matrix and k={1,⋯,M}.
- Normalize the amplitude information of $H_{k}\in C^{N_{R}\times N_{T}}$ to the range of [0,1] by discrete standardization operation of the following transformation formula as [29]
  (6) $$\begin{array}{c} {\bar{H}}_{k}\left(i,j\right)=\frac{H_{k}\left(i,j\right)-\min\left\{H_{k}\right\}}{\max\left\{H_{k}\right\}-\min\left\{H_{k}\right\}} \end{array}$$
  where $i=1,\cdots ,N_{R},j=1,\cdots ,N_{T}$.

#### 3.1.2. Data Labeling

To select the optimal CBAS antenna index of $H_{max}\in C^{N_{R}\times L_{t}}$ from all possible combinations of S, we opt for using the channel capacity *C* as the key performance indicator to generate the multi-label corresponding to each training sample, i.e., the full MIMO channel matrix $H\in C^{N_{R}\times N_{T}}$. Moreover, for the sake of enhancing the performance of the multi-label generation process, the ergodic search-based optimal CBAS method is applied to obtain the optimal transmit antenna subset. The multi-label generation process is summarized in Algorithm 1, where $S_{k}$ is referred to as all the λ=NTLt possible subsets generated by ${\bar{H}}_{k}\in C^{N_{R}\times N_{T}}$, $C_{k,\tau }$ stands for the channel capacity of the selected channel matrix subset $H_{s_{k,\tau }}\in C^{N_{R}\times L_{t}}$ associated with the maximum MIMO capacity value of $C_{k,max}$, $b_{k}$ with a length of $N_{T}$ denotes the multi-label of ${\bar{H}}_{k}\in C^{N_{R}\times N_{T}}$.
**Algorithm 1** Multi-label generation process**Input:***M* initialized binary multi-label vectors $b_{k}={\left[0,0,\cdots ,0\right]}^{T}$, *M* pre-processed full channel matrixs ${\bar{H}}_{k}\in C^{N_{R}\times N_{T}}$ 1:Initialize k=12:**while**k≤M**do**3:    Generate $S_{k}=\left\{s_{k,1},s_{k,2},\cdots ,s_{k,\tau },\cdots ,s_{k,\lambda }\right\}$ for ${\bar{H}}_{k}\in C^{N_{R}\times N_{T}}$4:    Calculate $C_{k,\tau }$ of $H_{s_{k,\tau }}\in C^{N_{R}\times L_{t}}$ according to (4) and (5)5:    Find $s_{k,max}$ in $S_{k}$ corresponding to $C_{k,max}$ and max={1,⋯,λ}6:    Set the corresponding position in multi-label vector $b_{k}$ to 1 according to $s_{k,max}$7:     k=k+18:**end while**
**Output:***M* multi-labeled vectors

More specifically, the multi-label used in our proposed MLCNN scheme is a vector consisting of $N_{T}$ binary bits. Each binary bit in the vector corresponds to an antenna. The binary bit of value 1 indicates that the corresponding antenna is selected, while value 0 means not selected. By contrast, the general single-label used in ML scheme is a vector composed of multiple binary bits [22] with the length of the vector equal to the total number of AS combinations of the ergodic search process, with bit value 1 corresponding to an antenna combination, noting that in single-label scheme, only one binary bit in the vector equals to 1. Table 1 shows a comparison example of our multi-label and the general single-label corresponding to $N_{T}=4$ and $L_{t}=2$, where it may be seen that as the number of AS combinations increases, the multi-label scheme may achieve significantly lower complexity than that of the conventional single-label ML schemes.

#### 3.1.3. MLCNN Model

**Figure 2:**
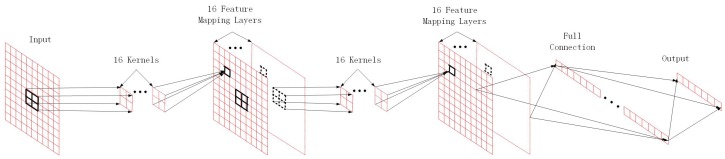
Proposed architecture diagram of MLCNN.

The general architecture of the proposed MLCNN model is illustrated in Figure 2, which is consisted of four main layers, namely the input layer, the convolution layers, the full connection layer, and the output layer. The Adam optimization method with initial learning rate of 0.0001 is adopted, and the binary cross-entropy is used as the loss function. The specific architecture of MLCNN is summarized in Table 2, where (None, 1, $N_{R}$, $N_{T}$) specifies that the size of input matrix is $N_{R}$ × $N_{T}$ and the number of channels is 1. num and ratio denote the number of neurons and dropout ratio, respectively, and both parameters can be adjusted according to the training situation. The dropout ratio is generally set as [0.1,0.5]. The construction and training of the MLCNN model is implemented in the framework of Keras.

It may be seen from Table 2 that the most significant difference between the proposed MLCNN model and the conventional CNN model is that MLCNN has no pooling layer in the convolution layers. This is because that the main functions of the pooling layer employed in conventional CNN models are down sampling, dimensionality reduction, redundant information removal, compression features and over fitting reduction [31]. These functions may be useful for processing the highly redundant data processing, i.e., image processing. However, in AS-aided MIMO IoT systems, CNN model is used for processing the MIMO CSI data, and each element in the MIMO channel matrix corresponds to a specific physical channel. Eliminating any data from the full MIMO channel matrix may destroy the structure of MIMO channel. As a result, the pooling layer is avoided in the proposed MLCNN model for AS-aided MIMO systems. Additionally, for the sake of fully extracting the characteristics of full CSI matrix $H\in C^{N_{R}\times N_{T}}$ in MLCNN, we generate 160,000 training samples and equally divide training samples into 4 groups. In the training process, we set the training batch size to 500 and use each group of training samples to train the model for 10 rounds.

### 3.2. Complexity Analysis

It was recognized that the overall training complexity of a CNN model is mainly imposed by the time complexity of the convolution layers, whereas the time cost of fully connected layers only contributes 5–10% computational time of the whole network. The online prediction time complexity of the network takes around 1/3 of the training time complexity. According to [32], the total training time complexity of all convolutional layers may be presented as

(7) $$\begin{array}{c} O\left(\sum_{l=0}^{d}n_{l-1}\cdot {s_{l}}^{2}\cdot n_{l}\cdot {m_{l}}^{2}\right) \end{array}$$

where *l* is the index of a convolutional layer, *d* corresponds to the number of convolutional layers. $n_{l}$ is referred to as the number of filters in the *l*-th layer, while $n_{l-1}$ is known as the number of input channels of the *l*-th layer. $s_{l}$ represents the spatial size of the filter of the *l*-th layer and $m_{l}$ is the spatial size of the output feature map of the *l*-th layer. It was previously mentioned that the overall delay of the AS-aided MIMO systems determined by the online AS prediction process. Therefore, we mainly focus on the overall online prediction time complexity of the proposed MLCNN algorithm, which may be calculated as

(8) $$\begin{array}{c} \frac{1}{3}\cdot O\left[\left(1\cdot 2^{2}\cdot 16\cdot N_{R}\cdot N_{T}\right)+\left(16\cdot 2^{2}\cdot 16\cdot N_{R}\cdot N_{T}\right)\right]\propto O\left(N_{R}\cdot N_{T}\right) \end{array}$$

LeNet model is a state-of-the-art data-driven AS method and was shown to be capable of operating in real time scenario [22]. As a comparison, the overall online prediction time complexity of LeNet model may be expressed as

(9) $$\begin{array}{c} \frac{1}{3}\cdot O\left[\left(1\cdot 3^{2}\cdot 32\cdot \frac{N_{R}}{2}\cdot \frac{N_{T}}{2}\right)+\left(32\cdot 3^{2}\cdot 64\cdot \frac{N_{R}}{4}\cdot \frac{N_{T}}{4}\right)\right]\propto O\left(N_{R}\cdot N_{T}\right) \end{array}$$

It may be seen that the prediction time complexity of the proposed MLCNN model is quite close to that of the LeNet model. Therefore, it may be concluded that the proposed MLCNN model can also operate in real time. At the same time, comparing the convolution layer structure of MLCNN model with that of LeNet model, we may find that our proposed MLCNN model structure has significantly fewer convolution kernels and smaller convolution kernel size than those of LeNet model structure, so the proposed MLCNN model structure needs far fewer training parameters than LeNet model structure does. Therefore, the proposed MLCNN model structure is easier to train than LeNet model structure.

## 4. Simulation Results

In this article, a quasi-static correlated Rayleigh fading environment was considered. The MIMO IoT system equipped with $N_{T}$ TAs and $N_{R}$ RAs, as well as employing $L_{t}$ transmit RF chains and $L_{r}=N_{R}$ receive RF chains, is denoted by $\left(N_{T},N_{R};L_{t},L_{r}\right)$. 256 neurons and the dropout ratio of ratio=0.2 were considered in the full connection layer. We considered (8,8;2,8) and (32,32;2,32) MIMO IoT systems and included the LeNet-based AS of [22] and NBAS of [7] as for performance comparison, where we employed same training data and training method to train LeNet model.

**Figure 3:**
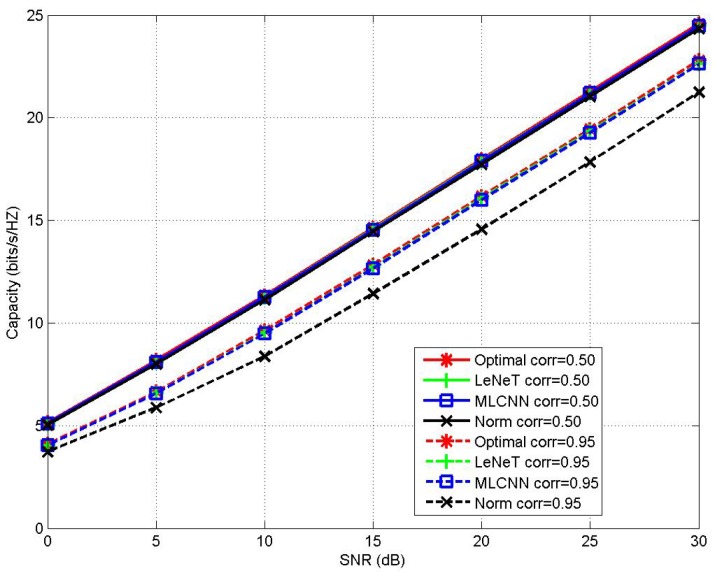
Channel capacity performance comparison between MLCNN-aided AS, LeNet-based AS and NBAS for (8,8;2,8) MIMO IoT system under correlation coefficients of corr=0.50and0.95.

**Figure 4:**
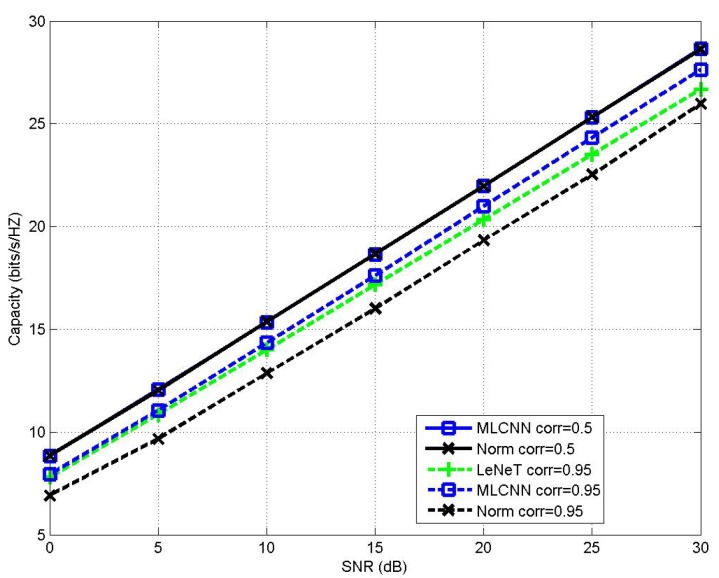
Channel capacity performance comparison between MLCNN-aided AS, LeNet-based AS and NBAS for (32,32;2,32) MIMO IoT system under correlation coefficients of corr=0.50and0.95.

The MIMO channel capacity performance of the proposed MLCNN-aided CBAS (8,8;2,8) MIMO IoT system under perfect CSI with the channel correlation coefficients of corr=0.50and0.95 is shown in Figure 3, in comparison to the performance of LeNet-based AS, NBAS and the optimal AS. It may be seen from Figure 3 that when corr=0.50, the above three AS algorithms were capable of achieving the optimal AS capacity performance. When the MIMO correlation coefficient increased to corr=0.95, both MLCNN and LeNet-based AS achieved the optimal capacity performance, while the NBAS experienced significant performance loss due to the increased channel correlation. More specifically, there showed around 1.42 bit/s/Hz performance gap between the NBAS- and MLCNN-based AS or LeNet-based AS at SNR of 25 dB. In addition, LeNet is a multi-class classification CNN model, which is not suitable for large-scale MIMO IoT system. This is because that the increase of antenna scale makes the total category space of LeNet-based AS significantly increase. To train this multi-class classification model for a large category space, we may need a larger training data set and a GPU with larger memory, or even a GPU cluster, which may be extremely costly and not be suitable for IoT applications. Unlike the classical LeNet, the proposed MLCNN is a multi-label classification CNN model, which achieve much lower labeling and training complexity in large-scale MIMO conditions. Take (32,32;2,32) MIMO IoT system as an example, the LeNet has a total category spaces of 322=496, and an output layer size of 496 as well, while the proposed MLCNN only needs an output layer of size 32. Figure 4 illustrates that the MIMO channel capacity performance of the proposed MLCNN-aided CBAS for (32,32;2,32) MIMO IoT system under the channel correlation coefficients of corr=0.50, in comparison to the performance of NBAS. It may be seen that when corr=0.50, the the proposed MLCNN-aided CBAS and NBAS achieved nearly the same capacity performance. Additionally, Figure 4 illustrates that the MIMO channel capacity performance of the proposed MLCNN-aided CBAS for (32,32;2,32) MIMO IoT system under the channel correlation coefficients of corr=0.95, in comparison to the performance of LeNet-based AS and NBAS, where the training samples were 160,000 for both proposed MLCNN and LeNet. It may be seen that when corr=0.95, MLCNN-aided CBAS achieved a significant performance gain of around 1.79 bit/s/Hz at SNR of 25 dB over NBAS and a significant performance gain of around 0.80 bit/s/Hz at SNR of 25 dB over LeNet-based AS. Experimental results of both Figure 3 and Figure 4 demonstrated that the proposed MLCNN-aided CBAS scheme was capable of outperforming the NBAS scheme under highly correlated MIMO channel environment, and was capable of outperforming LeNet in large-scale MIMO IoT systems with the aid of same number of training samples.

**Figure 5:**
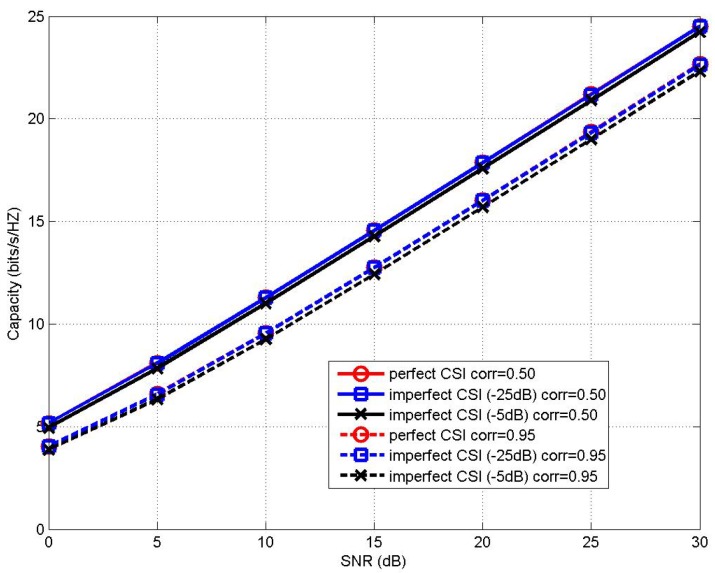
Channel capacity performance comparison between the proposed MLCNN in imperfect CSI and perfect CSI for (8,8;2,8) MIMO IoT system under correlation coefficients of corr=0.50and0.95.

**Figure 6:**
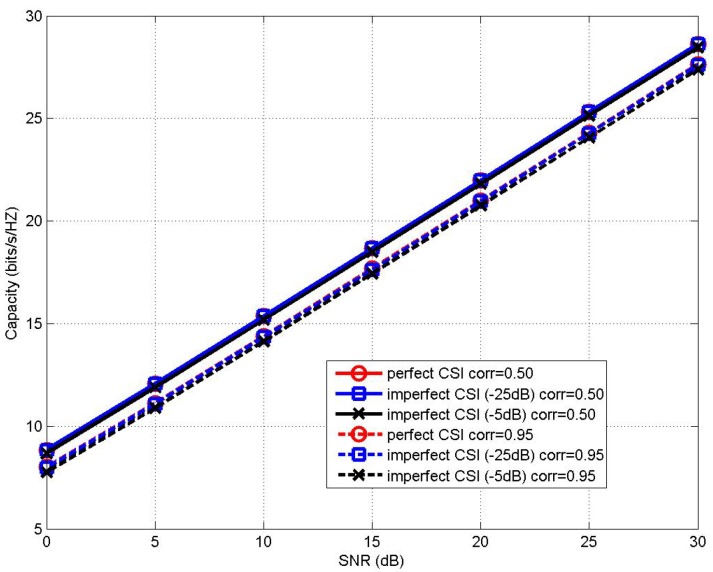
Channel capacity performance comparison between the proposed MLCNN in imperfect CSI and perfect CSI for (32,32;2,32) MIMO IoT system under correlation coefficients of corr=0.50and0.95.

The MIMO channel capacity performance of the proposed MLCNN-aided CBAS (8,8;2,8) and (32,32;2,32) MIMO IoT systems under imperfect CSI with the channel estimation error of −5 dB and −25 dB is shown in Figure 5 and Figure 6, respectively, in comparison to that of the perfect CSI case. It can be seen from Figure 5 that when corr=0.50and0.95, the capacity performance of MLCNN under imperfect CSI with channel estimation error of −5 dB only experienced a minor degradation of around 0.3 bit/s/hz compared to that of the perfect CSI case. In the case of channel estimation error of −25 dB, the proposed MLCNN scheme achieved almost the same performance of perfect CSI scenario. Similar phenomenon may be seen from the performance comparison of the (32,32;2,32) MIMO IoT system of Figure 6 as well. Therefore, it may be seen that the performance of the proposed MLCNN-aided CBAS algorithm is relatively insensitive to the effects of imperfect CSI.

## 5. Conclusions

We proposed a MLCNN-aided transmit AS scheme for end-to-end MIMO IoT communication systems under correlated MIMO channel environments. We adopted the simple yet efficient concept of multi-label in the proposed MLCNN-aided transmit AS MIMO IoT system, which may greatly reduce the length of training labels in the case of multi-antenna selection. Additionally, applying multi-label concept significantly improves the prediction accuracy of the trained MLCNN model under correlated large-scale MIMO channel conditions with less training data. The corresponding simulation results verified that the proposed MLCNN-aided AS scheme may be capable of achieving near-optimal capacity performance in real time, and the performance is relatively insensitive to the effects of imperfect CSI.

## Figures and Tables

**Table 1:** Example of Multi-Label and Single-Label comparison with $N_{T}=4$ and $L_{t}=2$.

Optimal Antenna Indices Combination	Multiple-Label	Single-Label
$s_{1}=\left[1,2\right]$	1100	100000
$s_{2}=\left[1,3\right]$	1010	010000
$s_{3}=\left[1,4\right]$	1001	001000
$s_{4}=\left[2,3\right]$	0110	000100
$s_{5}=\left[2,4\right]$	0101	000010
$s_{6}=\left[3,4\right]$	0011	000001

**Table 2:** MLCNN Architecture.

Layer	Architecture
Input layer	Pre-processed full CSI matrix $H\in C^{N_{R}\times N_{T}}$
Convolution layer1	data_format=’channels_first’
	batch_input_shape = (None, 1, $N_{R}$, $N_{T}$)
	filters = 16
	kernel_size = (2,2)
	strides = 1
	padding = ’same’
	Activation (’relu’)
Convolution layer2	data_format=’channels_first’
	filters = 16
	kernel_size = (2,2)
	strides = 1
	padding = ’same’
	Activation (’relu’)
Full connection layer	Flatten function
	num neurons
	Activation (’relu’)
	Dropout (ratio)
Output layer	$N_{T}$ neurons
	Activation(’sigmoid’)
